# Case of Tuberculous Disease

**Published:** 1842-03-12

**Authors:** Brodie S. Herndon

**Affiliations:** Fredericksburg, Virginia


*Case of Tuberculous Disease.* By Brodie S. Herndon, M. D.

The case of tuberculous disease, referred to as under treatment in my communication, which appeared in the Examiner of the 19th, presents the following history. The child, a girl of 4 years, was seized in October with cough, fever, and disordered bowels, and through the winter these symptoms continued with alternations of increase and diminution. The bowel complaint was in the form of dysentery, and constituted the principal part of her sufferings. The cough was continuous and dry; at one time there was excessive sighing; almost every breath was a gasp. The skin was excessively harsh and dry, and the emaciation progressive.

The physical signs (in which I am very indifferently skilled) revealed no cavity or extensive solidification; the lungs being pervious, and the respiratory murmur distinctly audible over every part of the chest, though louder than natural. The treatment consisted of mucilages, pectorals, warm baths, anodynes, clysters, and blisters. (I partake of Dr. Caspar Morris' dread of blisters in childhood, having often witnessed the most troublesome and distressing consequences from them.)

A week before death, which has taken place since my communication, hemiplegia of the right side occurred, and the final result was accompanied with convulsions of twenty hours duration.

*Examination of the body.*—Great emaciation. Brain,—about six ounces of water flowed from division of the membranes, and the ventricles contained about an ounce each. The body of the brain was healthy, and there were no tubercles. Chest,—numerous fine bridles of adhesions were found between the pleuræ; the lungs were filled with tubercles in the crude state, some of them just beginning to soften. They were most abundant in the clavicular regions; greater in the left lung; a portion of the organ at its most diseased point swam in water, and when washed seemed about half organ and half deposit. The bronchial glands were mere masses of tuberculous matter. Heart healthy; about four ounces of water in the pericardium. Abdomen,—peritoneum and omentum healthy; stomach and bowels pale and thin. From the rectum to the valve, the mucous coat of the large intestine was sprinkled with copious, distinct, circular tuberculous spots, in a state of ulceration. The small intestines comparatively exempt. Stomach and duodenum perfectly healthy. Liver sprinkled on its surface with whitish dots. Spleen mostly occupied with tuberculous matter; mesenteric glands very much loaded with it; kidneys healthy; uterine apparatus healthy.

*Fredericksburg, Virginia,* Feb. 23, 1841.

